# Supplementary material for: DMRforPairs: identifying Differentially Methylated Regions between unique samples using array based methylation profiles
Source: BMC Bioinformatics. 2014 May 15;15:141. doi: 10.1186/1471-2105-15-141 (PMC4046028; doi:10.1186/1471-2105-15-141)
Supplement: Additional file 2 — DMRforPairs output for the comparison of A431-MCF7 and NA17018-NA17105. Please start from the HTML files in each folder. Available via the BMC Bioinformatics website. [file 1471-2105-15-141-S2.zip › 1394847754114233_MOESM2_ESM/A431_MCF7/figures/30449.pdf]

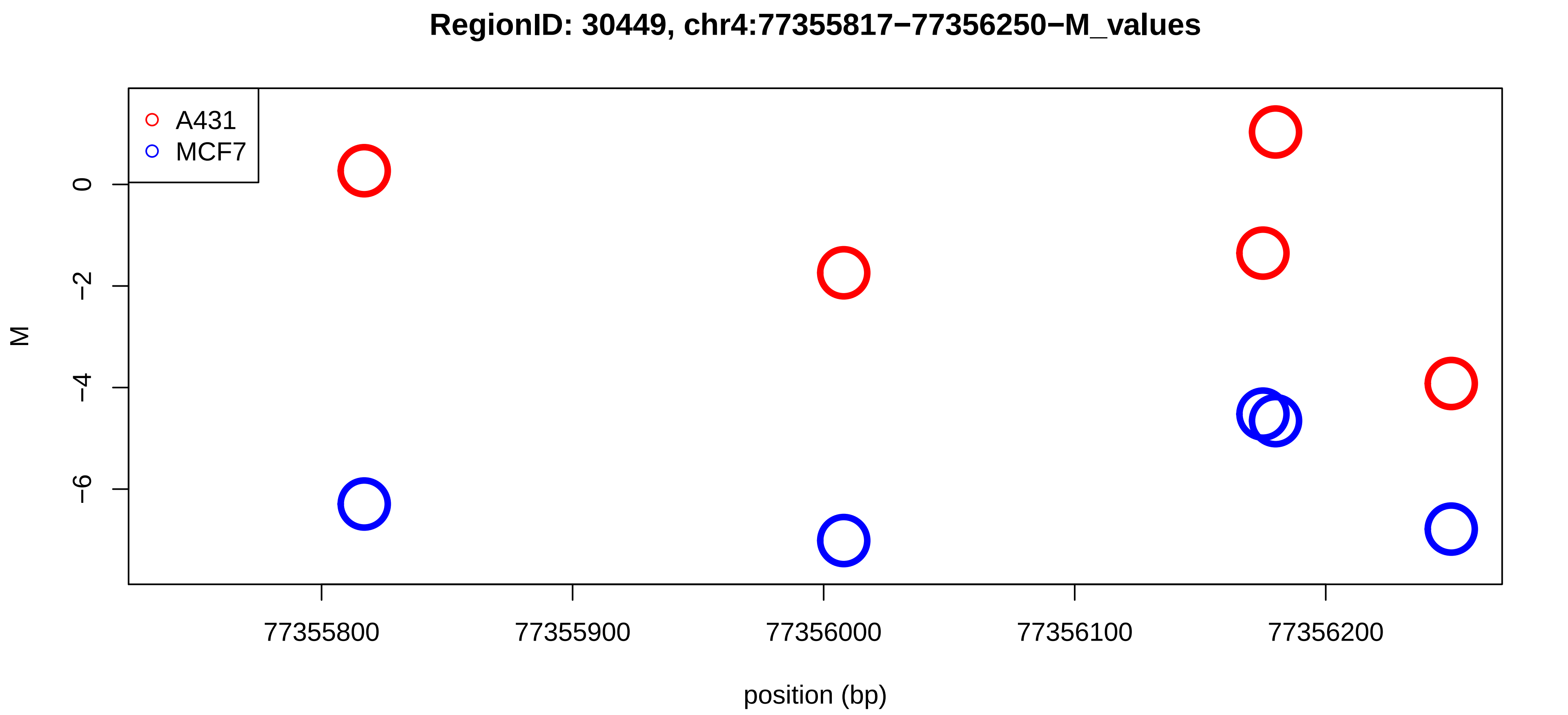
RegionID: 30449, chr4:77355817−77356250−M_values
A431
MCF7
0
−2
−4
−6
M
77355800
77355900
77356000
77356100
77356200
position (bp)

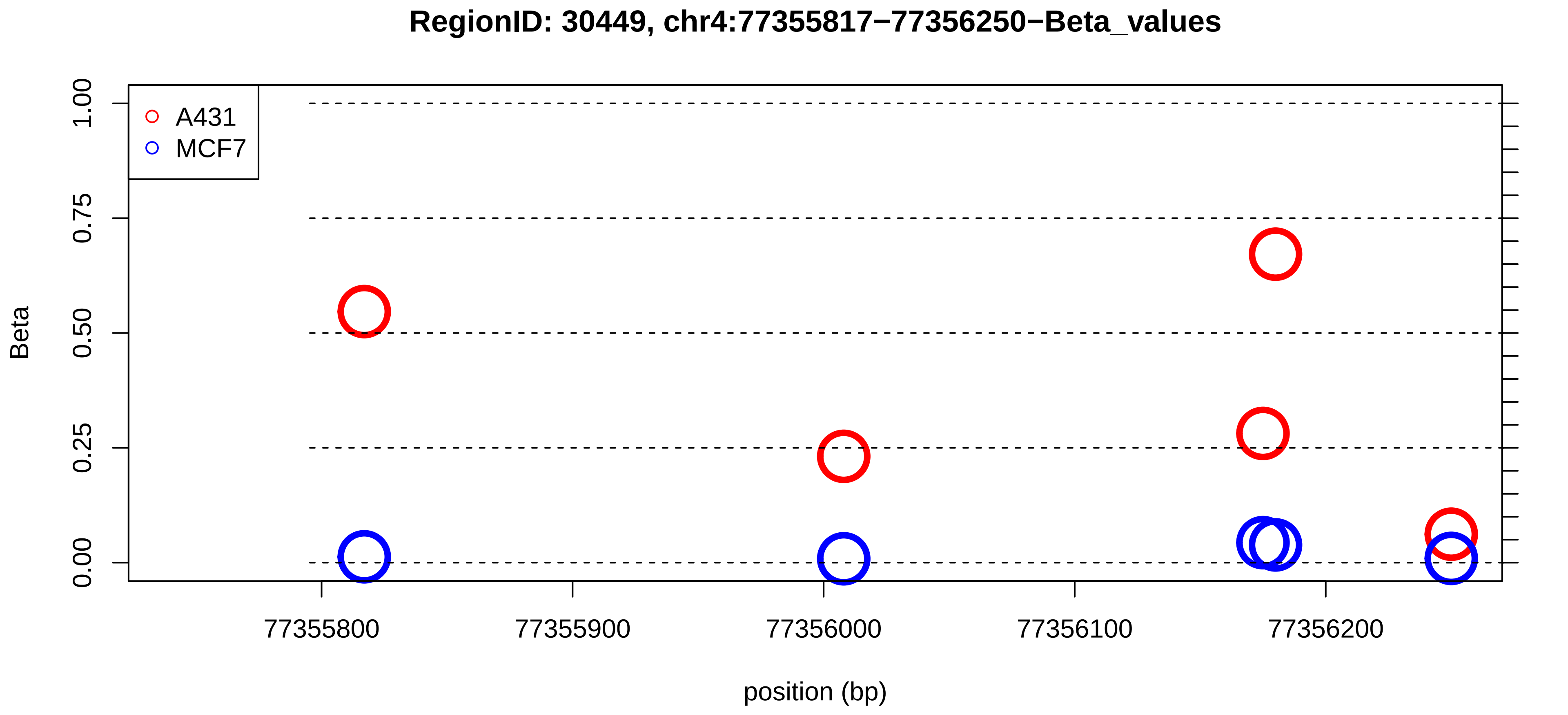
RegionID: 30449, chr4:77355817−77356250−Beta_values
A431
MCF7
1.00
0.75
0.50
0.25
0.00
Beta
77355800
77355900
77356000
77356100
77356200
position (bp)

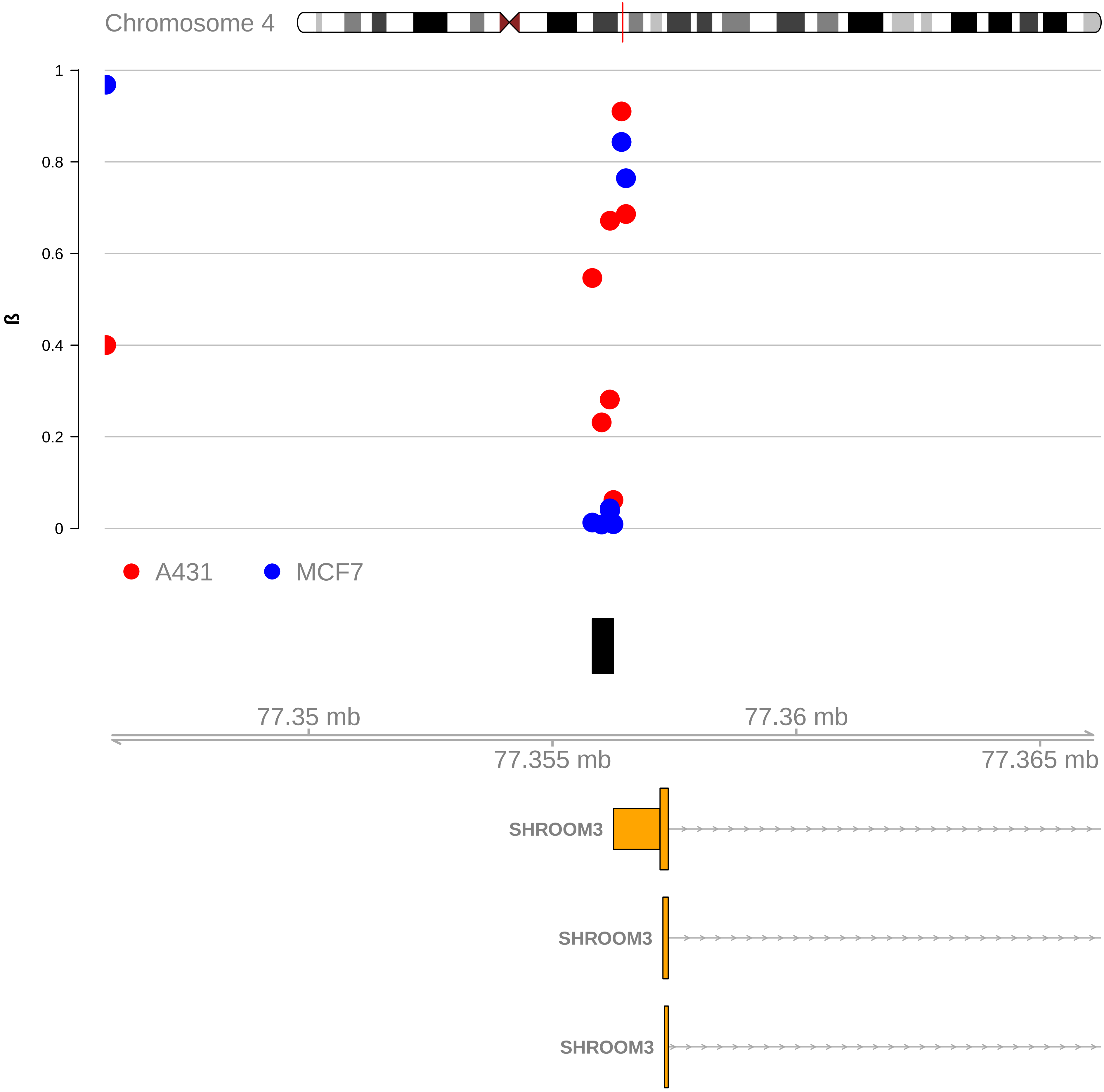
Chromosome 4
1
0.8
0.6
0.4
0.2
0
ß
A431
MCF7
77.35 mb
77.36 mb
77.355 mb
77.365 mb
SHROOM3
SHROOM3
SHROOM3
